# A model of collaboration between nursing education institutions in the North West Province of South Africa

**DOI:** 10.4102/curationis.v40i1.1670

**Published:** 2017-09-22

**Authors:** Kathleen K. Direko, Mashudu Davhana-Maselesele

**Affiliations:** 1 Agriculture, Science and Technology, North West University, South Africa; 2 Office of Rector, North West University, South Africa

## Abstract

**Background:**

Professional nursing in South Africa is obtained through a 4-year diploma offered at nursing colleges, or a 4-year degree in universities, and the South African Nursing Council (SANC) registered both for professional nursing. New SANC legislation now requires a bachelor’s degree for registration as professional nurse.

**Objectives:**

The aim of the study was to explore and describe perceptions of nurse educators and stakeholders to develop a model of collaboration for joint education and training of nursing professionals by colleges and universities through a bachelor’s degree.

**Method:**

A mixed methods approach was used to explore perceptions of nurse educators utilising a questionnaire, and perceptions of other nurse training stakeholders through interviews, about a model of collaboration between the college and the university.

**Results:**

Themes that emerged from the interviews included identifying collaboration goals, establishing a conducive environment, maximising exchange of resources, role clarification and perceived challenges. Quantitative results showed high agreement percentages (84.13%–100%) on most basic concepts and themes. A model of collaboration was developed indicating a framework, agents, recipients, procedure, dynamics, and terminus.

**Conclusion:**

A model of collaboration was acceptable to the majority of nurse education stakeholders. Other implications are that there was a need for the improvement of scholarship among nurse educators and clinical mentors, sharing rare skills, and addressing perceived challenges.

## Introduction

In South Africa, a professional nursing qualification is acquired through a 4-year diploma in nursing colleges or a 4-year degree in a university. Both were registered according to Regulation 425 of 1985 of South African Nursing Council (SANC) until new SANC qualifications, which require a bachelor’s degree at National Qualifications Framework (NQF) level 8 for professional registration. The change to a bachelor’s degree as a requirement for registration in professional nursing means that nursing colleges were to conform to requirements of higher education. The SANC had decided on 2018 as the last intake for the 4-year diploma to accommodate necessary changes to maintain an adequate production of professional nurses.

### Problem statement

Nursing colleges need to conform to higher education requirements to offer a bachelor’s degree for professional registration, as required by SANC. Phasing out the 4-year diploma and conforming to higher education requirements need time and could affect continuity in the production of professional nurses. Colleges produced most of the professional nurses in the North West Province (NWP): 259 as opposed to 91 from the universities according to SANC statistics, 2015. Researchers saw a need for collaboration between colleges and universities.

### Aims of the study

The aim of the study was to develop a model of collaboration between nursing education institutions (NEIs) in the NWP for offering a bachelor’s degree to produce professional nurses for the NWP. The goal of such a collaboration would be to provide an option for solving the problem perceived.

## Background

The *Nursing Act*, 1978 (Act 50 of 1978) as amended, accorded SANC full control of nursing education and training in the country (Searle 1988:149). Prior to the promulgation of the *Nursing Act* of 1978, training took place in hospital-based colleges to facilitate the integration of theory and practice (Searle 1965). According to Searle (1988), the De Lange Commission of Enquiry of 1980 had recommended the placement of colleges in post-secondary education. Based on this recommendation, the nursing colleges and universities were associated through formal affiliation agreements to monitor academic standards by 1986 (Mashaba 1995).

Section 2 of the *South African Qualifications Authority Act*, 1995 (Act 58 of 1995) classifies education and qualifications in the country into basic, further and higher education categories in a unified system, the NQF. All higher education institutions are placed under the National Department of Education according to the *Higher Education Act*, 1997 (Act 101 of 1997), by the *Constitution of South Africa* (Act 108 of 1996).

### Trends

Complex challenges and changes in healthcare and education require interdependence of available expertise, individual contributions, participation and pooled resources to maximise output and create synergy.

Cronenwett (2004) posits that the 21st-century nursing profession faces challenges that necessitate collaboration between academic and service entities. Increasing evidence shows that there is a direct relationship between better patient outcomes and nurse staffing levels, educational levels, and professional practice environments (Cronenwett, 2004). In the face of nursing staff shortages, both academic and practice entities have vested interests in increasing the supply of new nurses as well as faculty members who educate them.

O’Neil and Krauel (2004) also acknowledge the need to address nursing workforce issues through collaborative efforts because unilateral efforts have their limitations. They distinguish between two types of partnerships, namely vertical and horizontal (Williamson 1985, in O’Neil & Krauel 2004). The vertical partnership integrates functions along the continuum of production. In nursing, one might think of the vertical processes of recruitment of students, provision of education, recruitment to work, retraining as needed and development of new skills as required.

Horizontal partnerships, in contrast, involve aggregation between similar functions within an area (Doz & Hamel 1997, in O’Neil & Krauel 2004). Traditionally limited to geographic proximity, horizontal partnerships have been more possible in the advent of information technology. An example in nursing might be where nursing faculty in comparable programmes share their teaching strategies for similar courses or develop a common curriculum. Such partnerships often allow for elimination of duplication and improve efficiency.

O’Neil and Krauel (2004) observe that many successful entities have achieved partnerships across both vertical and horizontal axes, but that it is difficult to create both simultaneously. They further suggest five key things that nurse leaders must keep in mind when considering such partnerships.

These include development of a coherent institutional strategy; screening potential partners against the strategy; assessment of core competencies, assets, and weaknesses; advancement of mutually beneficial strategies; and structuring accountability to each other (O’Neil & Krauel 2004).

Primary healthcare development is impeded by weak strategic inputs, which could be improved by intersectorial collaborations to mobilise all stakeholders for cumulative experiences and evidence (Adeleye and Ngozi 2011)

Casey (2011) focuses on the need for collaboration to avoid the risk of exacerbating the ‘theory-practice gap’ in nursing. Like many contexts, the European Union has been pushing academic and service entities in nursing to move toward a more harmonised system where theoretical and practical learning are combined to produce more competent nurses.

Another trend is to resolve challenges related to curriculum and training issues. In some contexts educators are expected to train professionals for quality patient-centred care, effective team membership and moving the leadership and research agendas according to Herrin et al. (2006).

The SANC (2011) issued a circular for new qualifications, including a bachelor’s degree for registration as a professional nurse. The last intake for the 4-year diploma was extended to June 2015, and later 2018. While the 4-year diploma is being phased out, there is a need for the colleges to prepare for changes, and other NEIs to assist by closing possible gaps over the phasing out period and beyond. A closer cooperation of the NEIs could, through collaboration, continue adequate production of nursing professionals.

### The objectives

Objectives were to explore and describe perceptions of nurse educators and stakeholders about a collaboration between the college and the university in the NWP; identify and describe factors that would facilitate an effective collaboration between the NEIs in the province; and develop a collaborative model for training nurses according to new SANC qualifications.

### Definition of key concepts

Collaboration is work done jointly with others, especially in an intellectual endeavour (Freshwater & Maslin-Prothero 2005). In this study, *collaboration* refers to a joint endeavour for nurse education and training between the multi-campus nursing college, North West University (NWU) nursing departments, and other nurse training stakeholders in the NWP.

*The Higher Education Act* defines a college as any college established or declared as a college under the Act. In this study, college refers to the multi-campus nursing college in the NWP of South Africa.

Nurse educators are professional nurses who are qualified in teaching, and/or work in NEI of the NWP.

Nurse training stakeholders are officers managing nurse education matters in the *Department of Health*, the North West Province Department of Health (NWPDoH), the college, university and the clinical learning areas.

University refers to the North West University in the NWP of South Africa.

### Contribution to the field

This study contributes a model of collaboration between NEIs in the NWP based on the perceptions of nurse educators and other stakeholders and the relevant legal framework in South Africa. Literature assists in coping with and solving challenges because of change, such as conforming to higher education requirements. O’Neil and Krauel (2004) address workforce issues, and Herrin et al. (2006) address curriculum and training issues, as examples. Input from stakeholders and nurse educators contributes suggestions assisting policy-makers with appropriate decisions for a positive impact on all role players in the province and beyond.

### Literature review

Literature has shown that partnerships between academic institutions, healthcare organisations and governments help deal with varied challenges such as clinical learning, professional development, and policy changes in other countries (Hall 2009).

The purpose of the collaboration in this context was to facilitate working together of the NEIs, and other stakeholders to maximise the use of resources and skills for the production of professional nurses in the province. An effective collaboration between the nursing college and the university could facilitate maximisation of quality and quantity in nursing education for the province.

There are three case examples presented by the authors to challenge nurse leaders to be bold and think ‘out-of-the-box’ to work toward the full potential of nursing service to the public, instead of limiting themselves to ‘traditional’ forms of partnership (Bleich et al. 2004).

The first example was derived from a study by the Institute of Medicine (IOM 2003) in the United States. The report identified a number of important stakeholders in public health, including the community, healthcare delivery systems, employers and business, the media, academia and governmental/public health infrastructure. Given this analogy, authors challenged nurse leaders in academia to engage the entire continuum of stakeholders, not just hospitals or those that fell under the healthcare delivery system (Bleich et al. 2004).

In the second example, members of the University of Mississippi School of Nursing (USA) and the Sisters of Mercy-Vicksburg (USA) collaborated to plan and implement innovative initiatives to improve the health of rural citizens (Bleich et al. 2004).

In the third example, the University of Kansas School of Nursing (USA) faculty collaborated with the Cerner Corporation of Kansas City in order to advance clinical informatics in nursing education for baccalaureate and high-degree students. Together, they developed an information technology product that utilised relevant current clinical content (Bleich et al. 2004:285–294).

Collaborations between colleges and universities can be used for resolving training- and curriculum-related issues in nursing. In many contexts, nursing educators are challenged to train professionals who are not only competent in skills for quality patient-centred care but are also able to function effectively within a team and move the nursing research and leadership agenda forward. In a departure from diploma-based training, hospitals are progressively seeking to increase their nursing staff skills mix to include more bachelor’s-trained nurses. Collaborative partnerships were formed to address training-related challenges.

**FIGURE 1:**
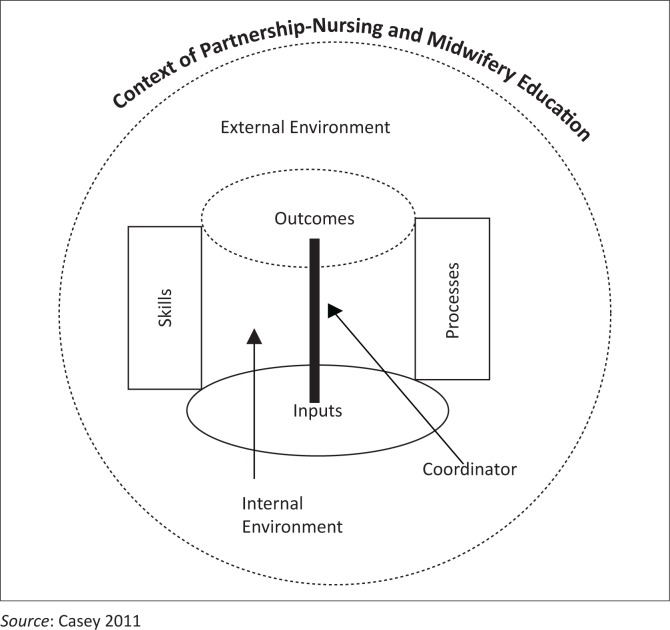
A new model for nursing and midwifery education.

Casey (2011) presents a variety of definitions for ‘partnership’ and ‘collaboration’ and points out that successful partnerships are non-hierarchical and partners share decision-making roles and have joint ownership of the resolutions of challenges. Further, the relationship involves commitment to improvement of efficiency. A framework for partnership developed from consultation with five nursing and midwifery education organisations in Ireland is presented in Figure 1.

Seven key concepts were identified as cornerstones of this partnership framework: *context, environment, inputs, processes, skills, outcomes,* and *the role of a coordinator.* The author argued that the role of a coordinator and partnership framework are crucial to the maintenance of collaborative relationships that bridge the theory/practice gap (Casey 2011:304–308).

In the United States, eight states have established mandated articulation agreements – which assure diploma graduates admission into a Bachelor of Science in Nursing (BSN) programme, establish the number of transfer credits to be accepted, and ensure consistency through the academic system. Twenty-four states have state-wide articulation agreements, where nurse educators/regulators/legislators have voluntarily established models of academic mobility for registered nurses (RNs) wishing to gain BSN training (Spencer 2008).

Collaboration is an important aspect of academic–clinical learning. Problem-based learning is the most innovative strategy to enhance problem-solving and prepares clinicians to cope in rapidly changing environments such as healthcare (Rakhudu, Davhana-Maselesele & Useh 2016)

In the Western Cape Province of South Africa, national policy imperatives prompted the need for consolidation, mergers, and a new way of approaching nursing education (Daniels 2010:42–48). This national imperative meant that institutions needed to collaborate to join the efforts of global and national transformation in education.

In addition, there was a need to increase operational efficiency and effectiveness so routine administrative and service functions were merged to pool resources and lower the costs. A project team consisting of representatives from all participating institutions developed a proposal for the development of a common teaching platform that encompassed an integrated planning framework and a Memorandum of Understanding (MOU).

Poor competencies among nurses in Malawi indicate a gap in theory and clinical practice. Varied academic–clinical partnerships improve competences and the healthcare system (Mvumbwe 2016).

The interdependence, contributions, participation and sharing of resources can be facilitated in a healthy collaboration with honest communication, mutual trust and respect, and common goals (D’Amour et al. 2005). The *Social Exchange Theory* was used by Gitlin, Lyons and Kolodner (1994) in a five-stage model to analyse collaboration. Basic assumptions of the *Social Exchange Theory* are ‘exchange’ and ‘negotiation’, and that social structures can be understood through an analysis of interpersonal transactions (D’Amour et al. 2005).

Theoretical assumptions of the *Social Exchange Theory* are that people will join a group or organisation that provides specific benefits or needs, and where they will, in turn, help to attain group or organisational objectives. Along with the exchange comes negotiation which continues to optimise benefits, reduce costs and move forward under conditions that will be fair to all stakeholders. Gitlin et al. (1994) expanded the *Social Exchange Theory* into a four-parameter model to include the following: exchange, negotiation, building an environment of trust, and role differentiation.

Collaboration between the college and university in the NWP could assist in improving quality and quantity in producing professional nurses, maximising the use of varied human and other resources and facilitating development in nursing education.

## Research method and design

A mixed methods approach which is mixing quantitative and qualitative data was used for this study (Creswell & Plano-Clark 2011).

### Design

**FIGURE 2:**
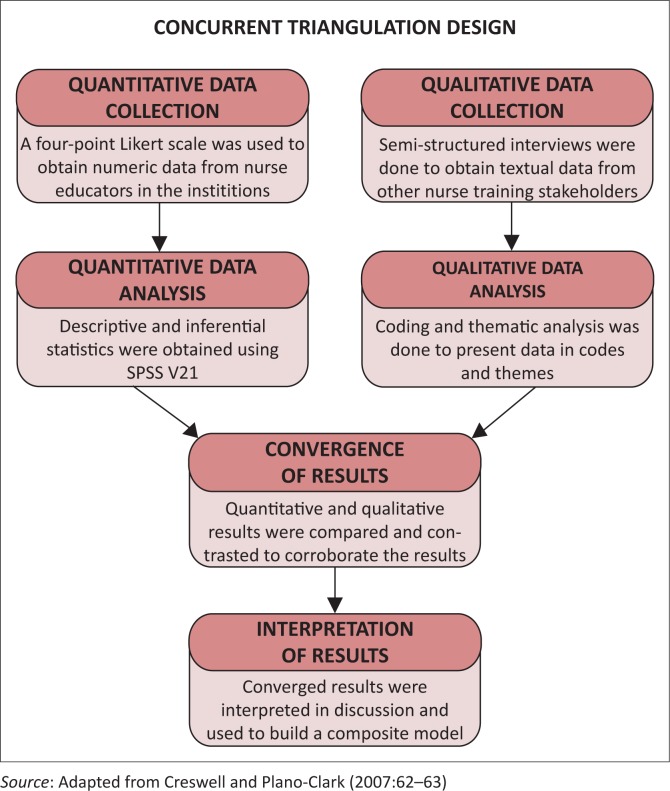
The convergence model of triangulation.

The convergence variant in triangulation design of mixed methods research was used. The purpose of the design was to combine different, but complementary data with the same purpose to understand the perceptions about collaboration, and to bring together the overlapping strengths and weaknesses of the quantitative and qualitative methods to obtain data. In the *Convergence Model of Triangulation* (Figure 2), qualitative and quantitative data have equal weighting, are collected and analysed concurrently, and results from both are compared, contrasted and merged for interpretation (Creswell & Plano-Clark 2011).

### Context of the study

The study was conducted in the nursing college and university campuses, and health service facilities of the NWP used by the NEIs for student placement.

### Sampling

**FIGURE 3:**
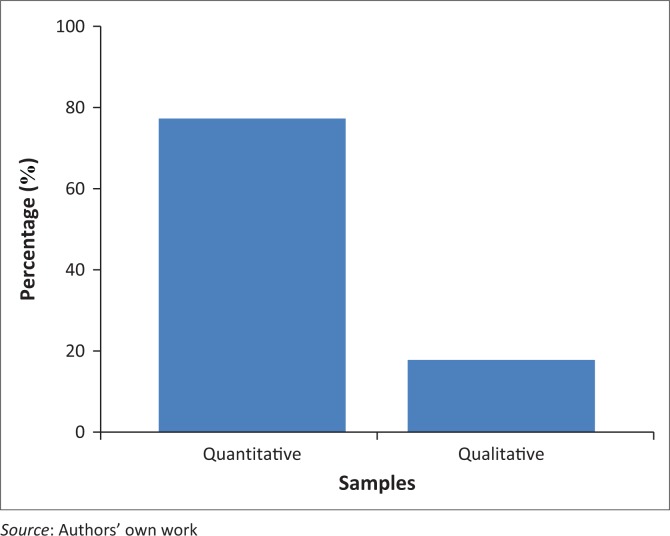
Percentage of participants in the quantitative and qualitative samples.

An all-inclusive population of 120 nurse educators who had been teaching for at least 1 year, and 15 purposely sampled stakeholders, who were leading officers in varied clinical facilities and North West provincial offices (Figure 3). A total of 68 questionnaires were received from nurse educators (57% compliance) and 15 stakeholders (100% compliance).

### Data collection method

Collection of data was concurrently done by the researcher. Semi-structured in-depth interviews were conducted at agreed appointments and venues with stakeholders to collect qualitative data, and quantitative data were collected using a questionnaire with a four-point Likert scale (four options indicating the extent of agreement or disagreement with a given statement). Questionnaires were sent to educators electronically and followed up with manual copies. The questionnaire and interview guide were based on the concepts of exchange, negotiation, role differentiation and building an environment of trust, from the *Social Exchange Theory*.

### Potential benefits and hazards

Benefits of the involvement in this study were opinion contributions for a possible solution to the challenge of policy changes for the benefit of the profession and the society. There were no hazards involved.

### Recruitment procedures

Participants were recruited by telephone and e-mail to offer their opinions freely, for the benefit of the profession and the society. They were entitled to withdraw at any stage if they chose to.

### Informed consent

Informed consent was provided in the form of an information sheet that participants could read and sign to indicate their consent to participate in interviews, and on a separate page of the questionnaire.

### Data protection

Data were protected by keeping it under lock-and-key where no one except the researcher could have access.

### Ethical consideration

Ethical approval and permission to conduct the study were obtained from the North West University (NWU-00070-12-AD) and the North West Province Department of Health. Permission (8/8/2013) to collect data was sought from campuses and clinical facilities involved. Informed consent was also sought from individuals interviewed and participants who responded to the questionnaires. Anonymity was upheld in both quantitative and qualitative data collection and safekeeping of data observed by the researcher. The protection of human rights was observed. Participants were not obliged to share what they chose to withhold. Interviews were conducted in privacy, and data were handled confidentially by the researcher. Interview transcripts were only identified by codes.

### Trustworthiness

Trustworthiness also refers to the degree to which the results of the research are truthful, authentic, accurate and credible. Measures to ensure trustworthiness are applied as shown according to the strategies in Table 1.

**TABLE 1:** Application of measures to ensure trustworthiness.

Strategy	Application
Credibility	Prolonged engagement with the participants was used by the researcher in presenting self for conducting the interviews. The inquiry was neutralistic, and enough time was given to the participant for self-expression, according to Brink, Van der Walt and Van Rensburg (2012). Non-verbal communication was observed at all times. Member checking was done by repeating conclusions and interpretations for the participants to confirm. Peer reviews were done by two experts in the field of qualitative research to ensure the value of truth.
Transferability	Triangulation was used by converging the qualitative and quantitative data which were collected concurrently, from different samples of participants. A dense description of data was supported by direct quotations from participants and a literature control to assess applicability (De Vos et al. 2011).
Dependability	The researcher spent time with the participants until there was saturation of information. Audiotape recording was done, and transcripts of the recordings and the tapes safely stored. Triangulation with concurrent quantitative data also promoted consistency. The peer review done by two experts in qualitative research added to dependability.
Confirmability	Neutrality was enhanced by triangulation of data according to the research design (Creswell 2012). Informed consent was obtained from the participants, and freedom of expression encouraged (Brink et al. 2006:119). The findings were supported by a literature control.

*Source:* From Polit and Beck (2008)

### Reliability and validity of quantitative data

The measuring instrument was found to be reliable, according to Cronbach’s alpha = 0.814 – which is a strong reliability. Sub-scale reliability is summarised in Table 2.

**TABLE 2:** Subscales reliability.

Scale	Cronbach’s alpha	Cronbach’s alpha based on standardised items	Number of items
Collaboration social construct	0.788	0.793	10
Negotiation construct	0.600	0.698	7
Role differentiation construct	0.609	0.637	5
Trust environment construct	0.874	0.877	10

*Source:* Adapted from Andale (2014)

Minimal difference indicates a good correlation.

### Data analysis

Themes and categories were used to analyse qualitative data. The number of occurrences of the codes or themes could be counted and entered in a spreadsheet to facilitate mixing with the quantitative data. SPSS version 21 software was used to obtain means and standard deviations to analyse and describe quantitative data from the questionnaire. Analysis of variance (ANOVA) was used to compare groups of educators from different NEIs or campuses. Frequency tables, pie charts and bar charts were used to summarise the demographics and responses. The two sets of data were converged for comparison, contrasting and interpretation through discussion (Creswell & Plano-Clark 2007).

## Results

Qualitative and quantitative results were collated, compared and contrasted. Basic assumptions of the *Social Exchange Theory*, as used by Gitlin and Lyons (2004), were utilised to facilitate comparison and contrasting of quantitative and qualitative results. The total number of participants for the quantitative and qualitative data sets was 80, consisting of 65 nurse educators (80%) and 15 representatives of clinical and administrative areas in nurse training (20%) (Figure 3).

**FIGURE 4:**
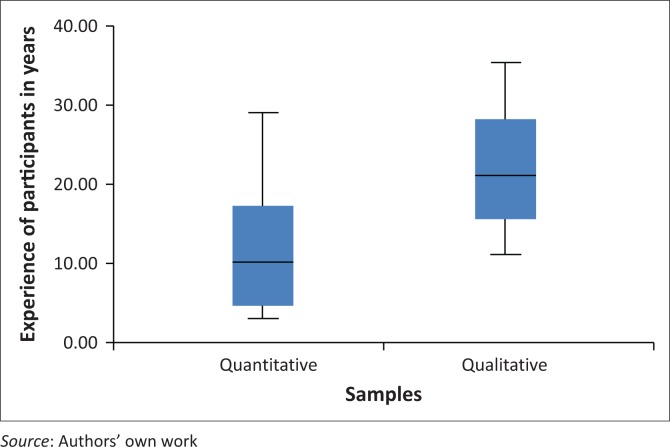
Experience in years of the participants in the quantitative and qualitative samples.

The mean experience of the nurse education stakeholders interviewed was almost double that of the nurse educators who responded to the questionnaires. The standard deviation in the experience was 8.70265 for the quantitative sample and 7.79722 for the qualitative participants (Figure 4).

The range of highest qualifications is the same between the quantitative and qualitative participants. There were a higher number of master’s degrees among nurse educators in the university and a higher number of bachelor’s degrees among educators in the college. The number of those with a diploma was less in the college and none in the university because of higher education conditions of appointment. The few diploma qualifications were found among those representing the other nurse training stakeholders.

### Quantitative results

Factors and perceptions that may influence collaboration between NEIs in the NWP.

This was acquired by a 4-point Likert-type questionnaire comprising a collaboration exchange construct, negotiation construct, role differentiation construct and trust environment construct. All responses were captured on SPSS V21 and to compare groups, *t*-test, ANOVA and Chi-square were used for cross-tabulations. Perceptions and collaboration facilitating factors for each construct of the *Social Exchange Theory* were indicated by percentages. Agreement levels of more than 50% can be accepted as positive responses, but if more than 50% strongly agree, the item concerned can be accepted as one that could facilitate collaboration, and one with disagreement at more than 50% could also be accepted as enhancing decision-making for the item concerned. Factors enhancing decisions for the collaboration are readily identified when they are strongly agreed or strongly disagreed with by more than 50%.

### Qualitative results

The following themes were identified:

Clarification of collaboration goals including implementation of SANC qualifications and improvement of stakeholder relationships:

‘Looking to the future in particular, where we need to come and implement these new qualifications, I believe collaboration is necessary.’ (Female, Master’s, Principal)

Establishment of a conducive environment including mutual trust, sound negotiation, political will and stakeholder commitment:

‘Communication, communication, communication! That’s my belief. Let’s create a trust environment to say “I’m not, I don’t have a hidden agenda”.’ (Female, PhD, HOD)

Maximising exchange of human and material resources:

‘Both institutions will benefit from the full potential in terms of resources.’ (Female, Bachelor’s, HOD)

Role clarification concerning responsibility and accountability:

‘Roles should be assigned according to capability, and outlined early so that people know what is expected of them, before they can take the responsibility.’ (Female, Bachelor’s, Tutor)

Perceived challenges such as fear, uncertainty and organisational culture:

‘You don’t only function as a nurse, but as part of a bigger picture and education landscape; such collaboration should not destabilise salaries like the “Occupation Specific Dispensation” did, instead of addressing the problems it was supposed to.’ (Male, Bachelor’s, Tutor)

### Convergence of results

The quantitative results were stated and followed up by statements from the qualitative results to compare and contrast findings. The high levels of agreement (100% to 53.97%) in all basic concepts of the *Social Exchange Theory*, were supported or confirmed by categories and subcategories of the themes developed in the qualitative results, which add to the validity of the results. Significant disagreements were clarified by comments from the respondents in the quantitative sample and also compared with statements from participants in the qualitative results. There is more agreement than disagreement in the comparison of results about the perceptions of stakeholders regarding collaboration between NEIs in the province.

**FIGURE 5:**
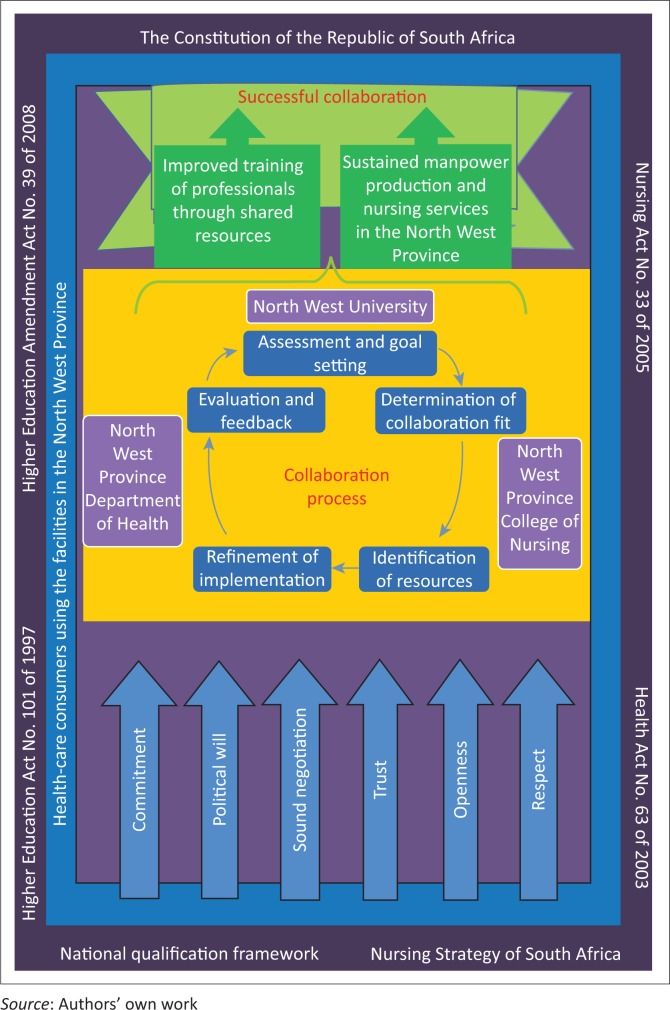
A schematic presentation of the collaboration model between nursing education institutions in the North West Province.

A model of collaboration (Figure 5) was developed using a five-stage process according to D’Amour et al. (2005). Quantitative and qualitative findings of the study were applied in the process. According to D’Amour et al. (2005), the five-stage process includes assessment and goal setting, identification of resources and expertise to share, determination of a collaboration fit for the situation, refinement of how the collaboration could be implemented and feedback to evaluate progress. The framework for model development can be viewed as a situation-producing theory, with a specified aim or goal, prescribed activities to attain the goal and a survey list guided by aspects of activity for the theory (Dickoff, James & Wiedenbach 1968). The survey list was related to aspects of activity in the model framework within a table (Table 3).

**TABLE 3:** Activity aspects as related to the framework model of collaboration.

Survey list	Question addressed	Collaboration model
Agents	Those responsible to guide the collaboration.	Leaders in the DOH, NWDoH, NWU and community representatives.
Recipients	Beneficiaries of the collaboration.	Learners and nurse educators at NEIs of the NWP, clinical service facilities and healthcare consumers.
Procedure	The process to guide the progress of the activities.	The five-stage process of the *Social Exchange Theory*.
Dynamics	Factors that will maintain the process.	According to participants, Commitment, Trust, Openness, Sound negotiation and Political will.
Framework/context	The context necessary to prescribe and guide the operation of the collaboration.	The Constitution of South Africa: *The National Qualifications Framework Act* 67 of 2008, *The Higher Education Act* 101 of 1997, *The Nursing Act* 33 of 2005, *The Health Act* 63 of 2003, The Nursing Strategy for South Africa, 2012 and Mandatory Agreements.
Terminus	Expected outcomes or goals of the collaboration.	Improved stakeholder relations and sustained manpower production and services.

*Source:* Information provided according to activity list by Dickoff et al. (1968)

DOH, Department of Health; NWDoH, North West Department of Health; NWU, North West University; NWP, North West Province.

### Description of the model

Figure 5 is a schematic representation of the model of collaboration between NEIs of the NWP, including the agents, who should guide the collaboration, the beneficiaries of the process that could be followed to achieve the collaboration, factors that could help to drive the process, the context necessary to support it and outcomes that should be expected from the collaboration.

### The agents

The responsibility of leading the process of developing collaboration between the NEI in the NWP can be given to leaders of the NWU, the NWPDoH and the NW college of nursing. These authorities participate in the current affiliation agreement between the university and the nursing college. The leaders of the institutions understand the needs of each stakeholder and how they could support each other.

The NWPDoH administers public health facilities used by the NEIs for student placement, and also administers and funds the college. The three structures are relevant to lead the development of a closer collaboration between them, as equal partners to achieve more than what they do currently. The agents are indicated in a circular diagram within the model to show equality.

### The dynamics

The stakeholders in the NWU, the NWPDoH and the North West college will need to maintain the process of collaboration in spite of challenges that might occur. According to the participants, commitment is essential from all stakeholders. There is a need for trust, respect and openness to continue sound negotiations during the process of collaboration. Political will from the government leaders will encourage progress and determination to achieve expected outcomes. In Figure 5 the above factors are shown as arrows impacting on the agents.

### The procedure

A five-stage process described by D’Amour et al. (2005) was utilised to develop collaboration, which will take time and daily efforts. The stages of the process are within the circle of the agents indicating engagement of the agents in the procedure from assessment and goal setting to evaluation and feedback. A cyclical repetition of the stages can be done during the process to advance the collaboration.

### The context

The development of the model of collaboration must occur within the context of legal, political and professional Acts and policies. In the schematic diagram of the model (Figure 5), the context is represented as a framework with all the relevant Acts and policies enclosing the agents, procedure, dynamics and collaboration outcomes which must be according to the context.

### The recipients

Beneficiaries of the collaboration will be students of the NEIs, and health consumers using the public facilities of the NWP for healthcare. In the model (Figure 5) they were placed in a framework within the context and surrounding the agents, process, dynamics and outcomes of the collaboration. Students should benefit from the efforts of all the stakeholders, and their success should benefit communities.

### Expected outcomes

Expected results of the collaboration are well-trained professional nurses, and sustained production of nurses for the NWP. The outcome is represented by a green ribbon above the agents, process and dynamics within the model. All aspects of the survey list as stated by Dickoff et al. (1968) were combined to form a schematic diagram of the collaboration between NEI in the NWP.

## Discussion

### Outline of results

#### Perceptions of nurse educators and training stakeholders about a model of collaboration between nursing education institutions in the North West Province

Stakeholders were 100% in agreement that the collaboration could facilitate professional and academic development, coordination of clinical learning placements and sharing of personnel with rare skills and improvement of qualifications through mentoring.

This objective was successful in a partnership between colleges and universities in Canada, where a comprehensive faculty development model was used as reported by Drummond-Young et al. (2010). The Chinese Ministry of Health accelerated nursing education baccalaureate and master’s degrees through international collaborations (Eddins, Hu & Liu 2011).

There was 98% agreement of stakeholders on the enhancement of community projects, partnering in research projects, development of teaching and learning skills, publications and coordination of recruitment and funding of students. Horns et al. (2007) reported the formation of a ‘clinical coordinating committee’ in a collaborative partnership to promote clinical placement, learning and projects.

There was opinion from stakeholders that sharing material resources should be limited to the convenience of proximity. Stakeholders felt that partners should not depend on the strengths of others as that would slow down progress and develop dependence.

There was a 98% agreement among the educators about expectation of new roles to suite the collaboration. Central and decentralised roles should be accommodated for the achievement of shared goals.

#### Factors that will facilitate an effective collaboration between nursing education institution in the North West Province

Responses from questionnaires and interviews were analysed to identify factors that could facilitate collaboration as perceived by the participants.

Leadership should be negotiated, and 84% preferred a decentralised structure because centralisation may induce fears of domination, while decentralisation ensures flexibility.

There was a 100% agreement on factors that contribute to an environment of trust to benefit the larger community beyond the collaborating partners.

According to Gitlin et al. (1994), the development of trust is critical to team building. Participants need to declare vested interests and avoid hidden agendas.

#### Developing a model of collaboration for nursing education institution in the North West Province

A theoretical framework was developed to guide the collaboration between NEIs in the NWP, and the model description given above is according to Dicoff’s survey list.

### Practical implications

The framework for a model of collaboration between the NEI in the NWP is a contribution to the use of the *Social Exchange Theory* in guiding or evaluating a collaboration process within a specific context, and provides an alternative decision to address the challenge of changed policies and common goals. The demographic information and perceptions explored can contribute to planning by stakeholders within the institutions of the NWP.

### Limitations of the study

The study was conducted only in the context of the NWP and may require adjustments for other provinces if replicated, because of differences in context. However, important lessons can still be learned from the study.

### Recommendations

There is a need to improve scholarship among nurse educators and clinical mentors, and to share rare skills. It is necessary to address perceived challenges ahead of policy changes.

Educators and facility managers should work jointly to improve the integration of theory and practice, and use opportunities for joint research to benefit practice and education.

Formal and informal interprofessional briefings, forums and meetings would benefit various parties. NEIs should be aware of clinical expertise needs of the facilities in order to align their plans to meet the needs.

## Conclusion

The objectives of the study were specified for the context of the NWP, but the results could provide lessons for other NEIs outside the province and the country, and beyond nursing. The study provides examples in methodology and theoretical frames for other research projects, and may also be suggestive of knowledge gaps for further research in nursing education within the NWP and beyond. One of the key recommendations of the Lancet Commission on Education of Health Professionals is to improve education by establishing networks and partnerships which take advantage of information and communication networks (Sharma & Zodpey 2013).

Examples include further investigation of perceived challenges of educators at the college about transition to the higher education sector, sharing rare skills and resources. NEI and facilities should jointly improve the integration of theory and practice and use opportunities for scholarship and joint research to improve clinical practice. Data from the interviews revealed eye-opening suggestions for teaching, service, community projects and other ideas.
